# Clinical and Prognostic Factors of Submandibular Gland Malignancies: A SEER-Based Analysis: Analysis of Submandibular Gland Tumors

**DOI:** 10.1007/s12105-025-01882-z

**Published:** 2026-01-21

**Authors:** Priya Arya, Logan W. Brown, Mikalah E. Maury, Kimberly B. Roth, David H. Posas, Robert M. Liebman, Matthew C. Ochsner

**Affiliations:** 1 Mercer University School of Medicine, 1250 E 66th St, Savannah, GA 31404 USA; 2 Department of Otolaryngology, Memorial Hospital University Medical Center, Savannah, GA USA

**Keywords:** Submandibular gland malignancy, Head and neck tumors, Benign, Malignant, Epidemiology

## Abstract

**Objective:**

While neoplasms of the submandibular gland (SMG) are rare, there is a nearly 50% risk of malignancy and its neoplasms are associated with poorer prognoses. Due to the rarity of these tumors, clinical and prognostic information relating to these tumors is limited; we aimed to elucidate these factors using an analysis of the Surveillance, Epidemiology, and End Results (SEER) database.

**Methods:**

A retrospective population-based analysis was performed with data regarding primary SMG malignancy from 2000 to 2019 as extracted from the SEER database. Kaplan–Meier curves and log-rank tests assessed differences in survival by tumor grade and treatment. To estimate odds of death, Royston-Parmar Proportional Odds models were used. Stratified analyses were conducted by tumor stage and histology subtype.

**Results:**

Data from 2875 patients were extracted. Kaplan–Meier analyses on a sample of 2809 patients showed survival differing significantly by tumor grade, histologic subtype, and treatment (*p* < 0.0001). Most favorable survival outcomes were amongst mucoepidermoid neoplasms, ductal and lobular neoplasms, and adenocarcinomas, and least favorable survival outcomes amongst epithelial and squamous cell neoplasms. Surgery alone and with adjuvant radiation and chemoradiotherapy both showed significantly lower odds of death for the overall sample.

**Conclusion:**

Multi-variate analysis indicated significantly lower odds of death with treatments involving surgery, while no significant differences were seen for radiation and chemoradiotherapy alone, likely due to the latter patients being poor surgical candidates or with advanced metastatic disease. Optimal treatment modalities for SMG malignancies are still being determined.

**Supplementary Information:**

The online version contains supplementary material available at 10.1007/s12105-025-01882-z.

## Introduction

Salivary gland malignancies encompass approximately 3–5% of head and neck cancers with 5–15% isolated from the submandibular gland (SMG) [1–4]. The preponderance of major salivary gland malignancies has steadily increased. Most are found in the parotid and submandibular glands and fewer cases reported within sublingual and minor salivary glands [1]. Relative to the parotid, SMG neoplasms have a rate of malignancy nearly twice as high and are associated with poorer prognoses [2].

Numerous exposures have been implicated in the development of salivary gland neoplasms, including modifiable factors such as alcohol and tobacco usage. Benign and malignant SMG neoplasms encompass a spectrum of histological types, and in most cases, surgical removal of the submandibular gland with possible adjuvant radiation therapy is the gold standard [3]. A neck dissection can be performed at the time of the initial surgery depending on the histology and stage. Other treatments in the literature include radiotherapy, chemoradiotherapy, and chemotherapy. Various factors contribute to survival including extent of lymph node involvement, local tissue invasion, and distant metastasis. However, information about these tumors' clinical and prognostic factors remains limited due to their rarity.

In recent years, few updates have been made synthesizing the epidemiology and prognostic factors of SMG neoplasms and various treatment paradigms [1–5]. We aim to analyze the Surveillance, Epidemiology, and End Results Program (SEER) database to assess outcomes of SMG malignancy patients and reveal any relationships between clinical or prognostic factors [6].

## Methods

### Data Extraction

Data was obtained using the SEER program databases (National Cancer Institute). The data from 22 registries encompassing incidence of diagnoses from 2000 to 2019 was utilized, encompassing 47.9% of the US population. Due to public availability of SEER and its deidentified data, this study was exempt from approval by the Memorial Health University Medical Center Institutional Review Board.

Participants were identified via the primary site cancer label of C08.0, malignant neoplasm of submandibular gland. Patient demographic variables included age groupings, sex, and race. Histopathologic characteristics included tumor grade, histology, tumor size, regional node involvement, and extent of spread. Clinical management and treatment data included months from diagnosis to treatment, surgery at the primary neoplasm site, radiation therapy, chemotherapy, combination and sequence of treatment, months of survival, and alive/dead status. Treatment arms were further categorized into the following: no/unknown treatment, surgery along, surgery plus adjuvant radiation and/or chemoradiotherapy, definitive radiation and/or chemoradiotherapy (i.e., the patient did not undergo surgery), and other treatments.

### Statistical Analysis

Kaplan–Meier curves and log-rank tests assessed differences in survival by tumor grade and treatment variables. To estimate odds of death, Royston-Parmar Proportional Odds models with 2 interior knots (3 d.f.) was used [7]. Royston-Parmer Proportional Hazards, Proportional Odds, and Probit models were considered, each with 0 to 5 interior knots. AIC (Akaike Information Criterion) and BIC (Bayesian Information Criterion) were used to compare model fit, and the model with the lowest BIC was chosen. Royston-Parmar models were chosen because preliminary statistical analysis revealed that the proportional hazards assumption was not met in the data. Therefore, Cox proportional hazards models were inappropriate. Statistical analysis was performed using Microsoft Excel and Stata software, version 18 [8]. *P* values < 0.05 were considered statistically significant.

## Results

### Demographics

The SEER database yielded 2,875 patients meeting the criteria of malignancy of the submandibular gland from 2000 to 2019. A total of 66 patients were excluded, leaving a final analytic sample of 2,809 (60 patients whose survival was recorded as 0 months, 6 patients whose survival was unknown). In this sample, 45.8% of patients were male and 54.2% were female. Age was reported within the database as 5-year groupings, and patients were grouped in the analysis into the following categories: under 50, 50–64 years of age, and 65 or older at 21.8% (n = 611), 29.5% (n = 830), and 48.7% (n = 1368), respectively. Demographic data are summarized in Table 1.

**Table 1:** Patient demographics

*Age, N (%)*	
< 50 years	611 (21.8%)
50–64 years	830 (29.5%)
> = 65 years	1368 (48.7%)
*Sex, N (%)*	
Male	1287 (45.8%)
Female	1522 (54.2%)
*Race, N (%)*	
Non-Hispanic White	1883 (67.0%)
Non-Hispanic Black	264 (9.4%)
Non-Hispanic Asian/Pacific Islander	322 (11.5%)
Non-Hispanic American Indian/Alaska Native	22 (0.8%)
Hispanic	303 (10.8%)
Unknown	15 (0.5%)

### Histopathologic Characteristics

Tumor grade was reported for 52.4% of the sample, with 6.9% Grade I tumors, 18.2% Grade II, 19.3% Grade III, and 8.5% Grade IV. The most common histology profiles, in order, were adenocarcinomas (42.3%), squamous cell carcinoma (18.3%), mucoepidermoid carcinoma (15.4%), complex mixed/stromal carcinoma (7.2%), epithelial carcinoma (6.9%), and ductal/lobular carcinoma (4.3%). Over 94% percent of tumor histology was encompassed within these five categories, and the remaining 5.5% were grouped as "other," including various rarer subtypes such as acinar, mucinous/serous, and soft tissue/sarcomas. Of the 64.8% of patients with reported extent of spread data, 29.2% of patients had a localized tumor, 12.2% had regional spread only by direct extension, 7.7% had regional spread both directly and by lymphatic extension, and 15.7% had regional lymph nodes involved or distant site(s)/node(s) involved. Histopathologic characteristics are summarized in Table 2.

**Table 2:** Neoplasm and treatment characteristics

Neoplasm characteristics	
*Main histological subtypes, N (%)*	
Adenocarcinomas	1189 (42.3%)
Squamous cell neoplasms	515 (18.3%)
Epithelial neoplasms	195 (6.9%)
Mucoepidermoid neoplasms	432 (15.4%)
Complex mixed and stromal neoplasms	203 (7.2%)
Ductal and lobular neoplasms	120 (4.3%)
Other neoplasms	155 (5.5%)
*Tumor grade, N (%)*	
Grade I, Well differentiated	193 (6.9%)
Grade II, Moderately differentiated	512 (18.2%)
Grade III, Poorly differentiated	542 (19.3%)
Grade IV, Undifferentiated/anaplastic	239 (8.5%)
Unknown	1323 (47.1%)
*Tumor stage at diagnosis, N (%)*	
Stage I, Localized spread only	821 (29.2%)
Stage II, Regional by direct extension only	342 (12.2%)
Stage III, Regional by both direct and lymph extension	217 (7.7%)
Stage IV, Regional lymph nodes or distant site(s)/node(s) involved	1 (15.7%)
Unknown/Unstaged	988 (35.2%)
Tumor size, N (%)	
< = 1 cm	142 (5.1%)
1.1–2.0 cm	556 (19.8%)
2.1–4.0 cm	862 (30.7%)
4.1–10.0 cm	454 (16.2%)
> 10.0 cm	12 (0.4%)
Unknown	783 (27.9%)
**Characteristics of treatment and modalities**	
*Months from diagnosis to treatment, N (%)*	
0–1 month	2235 (79.6%)
2–3 months	307 (10.9%)
> 3 months	72 (2.6%)
Unknown	195 (6.9%)
*Treatment performed, N (%)*	
No/unknown treatment	211 (7.5%)
Surgery alone	868 (30.9%)
Surgery + adjuvant radiation and/or chemotherapy	1476 (52.5%)
Definitive radiation and/or chemotherapy	143 (5.1%)
Other	111 (4.0%)

### Clinical Management and Treatment Information

Mean time from diagnosis to treatment was 19 days (± 35 days; data not shown). For the 93.1% of patients where months from diagnosis to treatment information was available, a total of 79.6% of patients were treated within 0–1 months following diagnosis, 10.9% were treated within 2–3 months of diagnosis, and 2.6% were treated in greater than 3 months following diagnosis. A total of 30.9% of patients underwent surgery alone at the primary neoplasm site, 52.6% underwent surgery with adjuvant radiation therapy and/or chemoradiotherapy, and 5.1% of patients had definitive radiation and/or chemoradiotherapy (i.e., no surgery). Only 7.5% of the sample had no or unknown treatment modalities. Mean time of survival was 69.6 months (± 65.2 months). Characteristics of treatment and modalities utilized within the study population are detailed in Table 2.

### Kaplan–Meier Survival Analysis

**Fig. 1:**
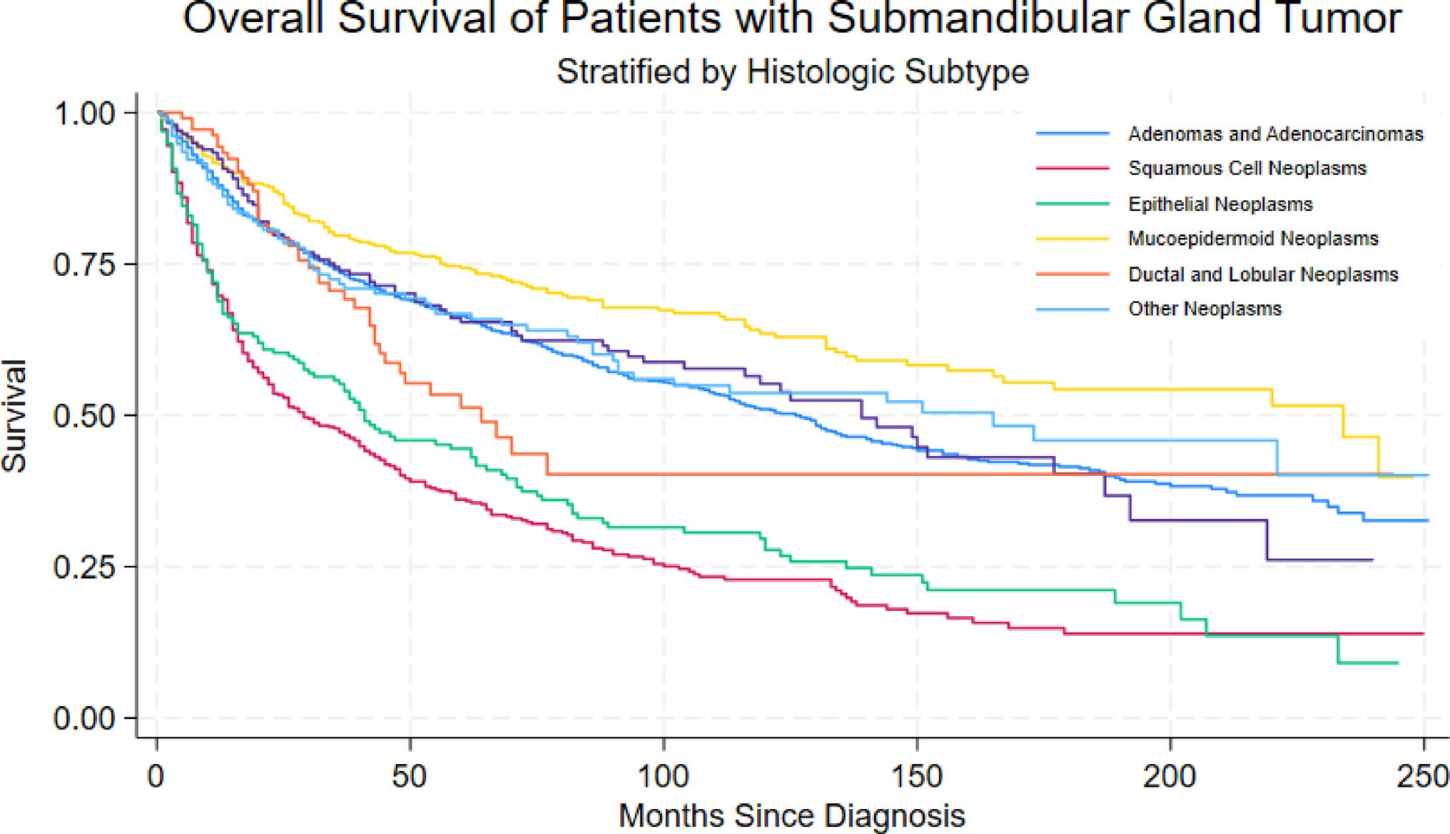
Survival outcomes of submandibular gland cancer by histologic subtype

**Fig. 2:**
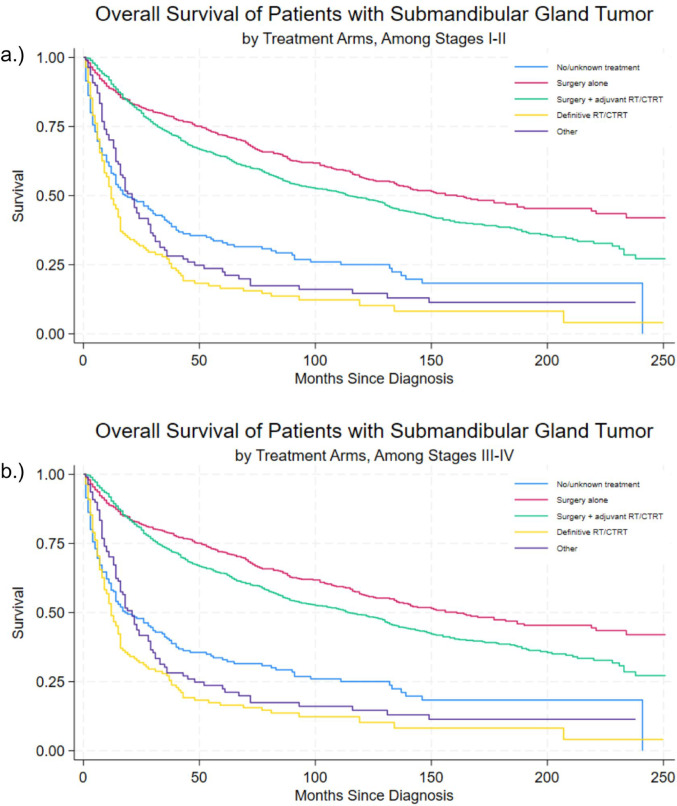
Survival outcomes by treatment arms, stratified by stage: **a** Among Stages I–II, **b** Among Stages III–IV. *Other includes any of the following: chemotherapy alone; sequence unknown, but both surgery and radiation were given; radiation both before and after surgery; radiation prior to surgery; and intraoperative radiation

A final analytic sample of 2,809 resulted in 1,374 deaths and a total of 16,299.6 person-years at risk. Kaplan–Meier analyses showed that survival differed significantly by tumor grade and treatment arms (all *p* < 0.0001). Figure S1 shows survival outcomes by tumor grade, which indicate similar outcomes for Grade I/II tumors and Grade III/IV tumors. Figure 1 shows most favorable survival outcomes amongst mucoepidermoid neoplasms, ductal and lobular neoplasms, and adenocarcinomas, and least favorable survival outcomes amongst epithelial and squamous cell neoplasms. Figure 2 shows survival estimates for the different treatment arms, stratified by staging: (a) stages I–II and (b) stages III–IV. Survival was improved with both treatments involving surgery, whereas no significant differences were seen with definitive and other types of treatment.

Overall and disease-specific survival is broken down by demographic and histopathologic factors in Table 3. Of note, many disease-specific survival outcomes were undefined, indicating 50% of the population was not yet deceased due to patients dying from causes other than SMG cancer. The overall survival of the study population was 81.1% after 1 year (n = 2277), 43.8% after 5 years (n = 1230), and 21.9% after 10 years (n = 616). For overall survival, male patients, non-Hispanic White patients, and 4.1–10.0 cm sized tumors were associated with poorer prognosis. In the non-parametric analyses stratified by stage, median survival time ranged from 9.7 years among those with Stage II to 2.2 years for Stage IV cancers (Stage I was undefined).

**Table 3:** Demographic and pathologic tumor factors and survival outcomes in months, median (SE)

Characteristic	Overall survival	Disease-specific survival
All patients	100 (5.4)	Undefined
*Age*		
< 50 years	Undefined	Undefined
50–64 years	15 (13.0)	Undefined
> = 65 years	53 (3.5)	197 (27.2)
*Sex*		
Male	66 (4.4)	Undefined
Female	139 (10.1)	Undefined
*Race*		
Non-Hispanic White	80 (4.6)	Undefined
Non-Hispanic Black	132 (25.9)	Undefined
Asian/Pacific Islander	234 (65.3)	Undefined
Non-Hispanic American Indian/Alaska Native	Undefined	Undefined
Hispanic	182 (21.9)	Undefined
*Histologic subtype*		
Adenocarcinomas	126 (7.0)	Undefined
Squamous cell neoplasms	29 (4.0)	66 (25.6)
Epithelial neoplasms	41 (7.6)	Undefined
Mucoepidermoid neoplasms	234 (25.8)	Undefined
Complex mixed and stromal neoplasms	139 (12.4)	Undefined
Ductal and lobular neoplasms	64 (9.0)	Undefined
Other neoplasms	165 (38.7)	Undefined
*Tumor grade*		
Grade I, Well differentiated	Undefined	Undefined
Grade II, Moderately differentiated	221 (23.7)	Undefined
Grade III, Poorly differentiated	39 (3.4)	66 (8.7)
Grade IV, Undifferentiated/anaplastic	47 (6.8)	81 (20.7)
Unknown	121 (8.1)	Undefined
*Tumor stage at diagnosis*		
Stage 1: Localized spread only	Undefined	Undefined
Stage 2: Regional by direct extension only	116 (9.3)	Undefined
Stage 3: Regional by both direct and lymph extension	33 (5.6)	45 (15.8)
Stage 4: Regional lymph nodes or distant site(s)/node(s) involved	26 (2.3)	Undefined
Unknown/unstaged	89 (8.7)	Undefined
*Tumor size, N*		
< = 1 cm	Undefined	Undefined
1.1–2.0 cm	Undefined	Undefined
2.1–4.0 cm	101 (10.6)	Undefined
4.1–10.0 cm	30 (4.1)	48 (10.6)
> 10.0 cm	53 (16.3)	53 (10.3)

Survival for a given treatment regimen across both overall study population and stratified by cancer stage are shown in Table 4. Median survival was shown to be the worst amongst patients with Stage III cancer who did not receive surgery (i.e., definitive radiation and/or chemoradiotherapy; 0.5 years). Survival outcomes for patients undergoing surgery were highly favorable compared to those who did not have surgical intervention. Figure 2 shows survival estimates for all treatment arms, stratified by staging: (a) stages I–II and (b) stages III–IV.

**Table 4:** Treatment regimens and survival in months, overall and by stage, median (SE)

Treatment	Overall survival				
	All stages	Stage I	Stage II	Stage III	Stage IV
No/unknown	18 (4.4)	34 (24.7)	4 (22.7)	137 (4.7)	13 (1.8)
Surgery alone	161 (18.1)	undefined	113 (7.6)	24 (9.2)	31 (10.5)
Surgery + adjuvant	114 (7.1)	undefined	130 (15.7)	41 (9.6)	42 (5.4)
Definitive radiation/chemotherapy	12 (1.4)	13 (21.0)	38 (6.0)	6 (5.4)	12 (0.8)
Other	21 (2.3)	149 (undef)	14 (6.7)	29 (7.1)	16 (1.5)

### Royston–Parmar Proportional Odds Models

Odds of death by individual treatment modality are detailed in Table 5, overall and stratified by histologic subtype (n = 2794 for the overall model, as 15 observations were dropped due to missing race/ethnicity). Surgery significantly lowered the overall odds of death for the sample, both alone and in combination with adjuvant therapy. It significantly lowered the odds of death for most histologic subtypes (except for complex mixed and stromal neoplasms, and ductal and lobular neoplasms). Definitive radiation and/or chemoradiotherapy was not significantly associated with survival in the overall sample or by histologic subtype. Estimates are adjusted for age, sex, race/ethnicity, histology subtype (in the overall model only), grade, and stage. Table S1 presents odds of death by treatment modality, stratified by staging categories. Regardless of staging, both surgery arms significantly decreased odds of death. Definitive radiation and/or chemotherapy as well as other types of therapy were unrelated to odds of death, regardless of staging.

**Table 5:** Odds of death overall and per histologic subtype by treatment modality

	Odds of death (95% CI)	*p* value
*Overall*		
Surgery alone	0.23 (0.17–0.32)	< 0.001
Surgery + adjuvant RT/CTRT	0.22 (0.16–0.30)	< 0.001
Definitive RT/CTRT	1.15 (0.76–1.72)	0.511
Other	0.68 (0.44–1.05)	0.083
*Adenocarcinomas*		
Surgery alone	0.25 (0.14–0.47)	< 0.001
Surgery + adjuvant RT/CTRT	0.18 (0.10–0.32)	< 0.001
Definitive RT/CTRT	1.55 (0.69–3.47)	0.292
Other	0.54 (0.25–1.16)	0.114
*Squamous cell neoplasms*		
Surgery alone	0.38 (0.21–0.69)	0.001
Surgery + adjuvant RT/CTRT	0.26 (0.15–0.45)	< 0.001
Definitive RT/CTRT	1.14 (0.61–2.11)	0.685
Other	0.62 (0.28–1.35)	0.229
*Epithelial neoplasms*		
Surgery alone	0.17 (0.06–0.44)	< 0.001
Surgery + adjuvant RT/CTRT	0.22 (0.09–0.52)	0.001
Definitive RT/CTRT	0.72 (0.26–2.00)	0.529
Other	0.72 (0.21–2.43)	0.594
*Mucoepidermoid neoplasms*		
Surgery alone	0.19 (0.05–0.67)	0.010
Surgery + adjuvant RT/CTRT	0.46 (0.13–1.65)	0.232
Definitive RT/CTRT	0.95 (0.19–4.69)	0.946
Other	0.85 (0.12–6.14)	0.872
*Complex mixed and stromal neoplasms*		
Surgery alone	0.58 (0.05–6.50)	0.655
Surgery + adjuvant RT/CTRT	1.01 (0.09–11.40)	0.994
Definitive RT/CTRT	19.09 (0.38–950.54)	0.139
Other	1.30 (0.08–21.98)	0.854
*Ductal and lobular neoplasms*		
Surgery alone	1.31 (0.13–12.85)	0.814
Surgery + adjuvant RT/CTRT	0.63 (0.08–4.94)	0.657
Definitive RT/CTRT	0.00 (undefined)	0.987
Other	0.00 (undefined)	0.988
*Other neoplasms*		
Surgery alone	0.08 (0.02–0.25)	< 0.001
Surgery + adjuvant RT/CTRT	0.07 (0.02–0.23)	< 0.001
Definitive RT/CTRT	0.26 (0.05–1.36)	0.110
Other	0.65 (0.13–3.15)	0.593

Estimates are adjusted for: age, sex, race/ethnicity, histology subtype (in the overall model only), grade, and stage. The reference category for all estimates is no/unknown treatment

*RT* radiation therapy, *CTRT* chemotherapy

*n = 2794 for the overall model. 15 observations were dropped due to missing race/ethnicity

## Discussion

The submandibular gland accounts for a small fraction of salivary gland tumors. Previous studies estimate approximately half of SMG tumors as benign and half as malignant [8]. Due to their low occurrence rates, the current literature is limited on epidemiology, rates of histological subtypes, and survival. The SEER database lends a greater sample size with data that has been employed to examine survival for other malignancies. Multi-variate analysis was also able to find multiple independent correlations with overall survival including demographics, histological subtype, grade, stage at presentation, size, and treatment modality. Another study conducted by Lee et al. examined survival for SMG tumors using SEER data from 1973 to 2011 and discussed correlates with size, stage, and type [2]. Our study updates and builds upon this work by assessing how survival is impacted by treatment modality for different histological subtypes and tumor grades.

The six reported subtypes in our analysis comprised over 94% of sample within the SEER database. The additional “Other” category included some other rarer subtypes such as acinar, mucinous/serous, and sarcomas/soft tissue tumors. Adenocarcinomas made up the majority of reported SMG tumors, though we suspect that this is including adenoid cystic carcinoma which is the most common histology reported in the literature, followed by squamous cell carcinoma and mucoepidermoid [10]. Previous studies similarly show a preponderance of adenoid cystic carcinoma, squamous cell carcinoma, and mucoepidermoid carcinoma, with rates in the literature reporting 36%, 18.1%, and 16.9%, respectively [2, 9, 11]. Notably, primary squamous cell carcinoma was previously considered rarer due to the infrequent occurrence of squamous metaplasia of the ductal epithelium [12]. It is impossible to know whether the diagnosis of squamous cell carcinoma of the SMG is in fact malignancy generated within the parenchyma of the gland itself, or possibly a metastatic lymph node adjacent to the SMG, despite the selected code including only primary SMG malignancies. Survival for reported histologic subtypes in our study showed highest rates amongst mucoepidermoid neoplasms, ductal and lobular neoplasms, and adenocarcinomas, and lowest rates amongst epithelial and squamous cell neoplasms. In the current literature, adenoid cystic carcinoma has been shown to have the highest median overall survival at approximately 8–12 years, whereas squamous cell carcinoma exhibited the lowest at 1–4 years [2]. Our research is therefore in line with previously reported rates of malignant transformation regarding various histological subtypes, as well as the aggressiveness of squamous cell cancers relative to other subtypes [12].

Generally, salivary gland tumor incidence increases with age. Ord et al. found malignant submandibular tumors were more common in the 6th decade of life, with the median reported age to be around 55 [12]. This aligns with our results, as almost 30% of those diagnosed were within the 15-year window from 50 to 64 years of age. In addition, almost half of those diagnosed were over 65 with the highest prevalence, in order, among the 7th, 8th, and 6th decades of life. Since a greater percentage of SMG tumors are malignant, they are likely more common in older adults who experience compromises in immune function and increased lifetime exposure to carcinogens. However, our survival analysis found the age group from 50–64 had a lower overall survival compared to patients over 65. Our sample was 54.2% female, deviating slightly from previous study showing a slight predilection for males [2]. Our sample indicated that women had overall survival rates approximately double that of men, similar to Lee et al. who also found female sex as beneficial to survival. In other cancers, it has been documented that women have better overall survival compared to men due to a variety of factors outside of tumor characteristics including lifestyle differences and prevalence of additional comorbidities, however, influences of this sex-based disparity remain unknown [13, 14]. Lastly, our study found race indicating differences in overall survival, with non-Hispanic White patients having poorer overall survival relative to Asian, Black, and Hispanic counterparts. This could also be influenced by numerous factors such as presence of additional co-morbidities or delays in time to treatment.

Survival curves by grade indicate similar outcomes for Grade I/II tumors and Grade III/IV tumors. This lends credence to the idea of tumor grade consolidation into two designations that have been previously used in the literature: low- and high-grade. With regards to low-grade salivary malignancies, one study showed 98.9% 5-year survival and 97.4% at both 10-year and 25-year survival with T1/T2 staging of mucoepidermoid carcinoma [15]. Previously specified histologic subtypes within the low-grade category included acinic cell carcinoma and low-grade polymorphous adenocarcinoma [12]. This aligns with our results indicating adenocarcinomas as well as mucoepidermoid carcinomas as having improved survival. High-grade malignancies include salivary duct carcinoma, carcinosarcoma (malignant mixed tumor), squamous cell carcinoma, oncocytic carcinoma, and small and large cell carcinoma. These cellular profiles are associated with high-risk treatment failure, prompting aggressive multimodality approach [12]. Prior literature has showed hazard of death was significantly increased with higher-grade tumors, particularly among Grade III and IV neoplasms. Haderlein et al. found that high tumor grade is a highly significant predictor not only for a shorter metastasis-free survival, but also for a shorter overall survival [16]. This supports our results, as our analysis showed high-grade epithelial cell and squamous cell neoplasms to have the worst survival.

Spread of disease is another important prognostic factor, with some studies having identified cervical lymph nodes as the best estimator of severity for patients with SMG cancer [4]. Rates of metastasis varied in the literature, ranging from 20 to 50%. [17–19] Bradley et al. found that rate of distant metastasis in salivary gland cancer ranges from 20 to 40% with a higher rate in high-grade tumors [20]. Other retrospective studies have reported a high rate of distant metastases in high-grade tumors [21, 22]. This may inform lower survival rates in higher-grade tumors, as these patients were more likely to already have distant metastasis at time of diagnosis.

Treatment recommendations for salivary gland malignancies are typically extrapolated from parotid tumor data as follows: surgical resection of malignancy primarily up-front followed by pathology driven adjuvant radiation with the potential to add chemotherapy sensitization. Thus, there remains an opportunity to create informed treatment guidelines for SMG tumors specifically, as the behavior of parotid and submandibular gland tumors can vary, with higher rates of malignancy up to 30% in submandibular neoplasms [11, 17]. Differentiation between benign and malignant lesions is challenging using clinical assessment alone; therefore, histological diagnosis is done either via initial whole tumor resection or via fine-needle aspiration cytology under ultrasound guidance. While needle biopsies can aid in treatment planning and limit additional unnecessary surgery, the higher preponderance of SMG tumors for malignancy should lead to caution and a lower threshold when deciding to undergo whole tumor resection as the primary treatment plan.

Those receiving surgical intervention usually have a less invasive burden of disease with higher likelihood of curative treatment, providing rationale to those undergoing surgical therapy having lower hazard ratios. The mainstay treatment for submandibular gland malignancies is surgical management. Survival outcomes may vary depending on the surgical approach, including the completeness of tumor resection and whether a concurrent neck dissection is performed. Standard management of surgically resected, high-grade carcinoma includes surgical excision of the primary site, as well as neck dissection [12]. Postoperative adjuvant radiation is recommended in high-grade salivary carcinoma, and in cases of close or positive margins [12]. Lower-grade tumor diagnoses generally do not require elective neck dissection [23].

Definitive chemotherapy is mainly restricted to locally recurrent or distant metastases and may not improve survival; it is usually employed as a last-line effort [17, 24–27]. Those receiving systemic chemotherapy may have had more locally advanced disease or frank distant metastasis making them sub-optimal surgical candidates. Additionally, having larger, more irregular, and poorly differentiated tumors predisposes to less favorable outcomes regardless of treatment modality. When indicated, postoperative chemotherapy usually includes drugs such as cisplatin. Another factor impacting survival is that among older patients, adjuvant chemotherapy/radiation therapy has been shown to carry more toxicity and mortality than radiation alone in comparison to younger patients [28]. The Kaplan–Meier curves in this sample indicated those receiving definitive radiation and/or chemoradiotherapy did not have an improved survival rate, likely due to higher-staged neoplasms with poorer prognoses in these patients. This is somewhat underscored by the results stratified by staging, where the point estimates indicate better survival among earlier stages and worse survival among stages III and IV, albeit both estimates are non-significant. Regarding radiation therapy, recommendations of postoperative radiotherapy include locally advanced tumor stage (pT3/T4), invasion of the bone, perineural spread, peri-nodal spread, close or positive resection margins, high-grade histology, and all adenoid cystic carcinomas except low-grade pT1 with wide margins [28–31].

The SEER database provides substantial data from a considerable number of patients. However, this study is not without limitations. Limitations include its retrospective nature, which restricts the data that can be leveraged to that which can be extracted from the database. Not all patients were able to be categorized by treatment sequence. Additionally, adenocarcinoma was the most common subtype. While our assumption is that this is including adenoid cystic carcinoma, which is known to be the most common histology subtype in submandibular gland pathology, it is not directly stated [10, 32]. Furthermore, this could include conventional adenocarcinoma in addition to acinic cell carcinoma. It is unknown the extent of reliability and consistency regarding reported attributes of each tumor if these neoplasms were characterized by numerous different pathologists. In the database, regarding each given treatment modality, patients were categorized as “yes” or “no/unknown” which did not allow for sub-stratification between patients who did not receive a given therapy versus those whose status was truly unknown. Additionally, some patients recorded in the database are lost to follow-up. Limited conclusions regarding the efficacy of differing management approaches can be drawn, and further investigation should include a prospective study with varying treatment paradigms in SMG malignancies. Finally, some subgroups contained relatively small sample sizes, leading to unstable or imprecise estimates in the odds of death.

## Conclusion

This study augments existing data regarding SMG pathology. In this sample, most favorable survival outcomes were observed amongst mucoepidermoid neoplasms, ductal and lobular neoplasms, and adenocarcinomas, and least favorable survival outcomes were seen amongst epithelial and squamous cell neoplasms. Multi-variate analysis indicated differences in survival by tumor grade, histology, and therapeutic modality, with odds of death shown to be lower with modalities involving surgery. This likely reflects selection of patients with earlier-stage disease, better performance status, and resectable tumors, in whom surgery is often combined with adjuvant radiotherapy with curative intent. In contrast, chemotherapy is more commonly administered to patients with advanced stage, poor performance status, or distant metastases, which may explain the higher observed odds of death in this group. Other types of therapy were generally not associated with odds of death, likely due to the latter patients often being poor surgical candidates with unresectable disease. Due to their uncommon nature, SMG malignancies need further prospective study to determine optimal management and prognosis.

## Supplementary Information

Below is the link to the electronic supplementary material.

Supplementary Material 1

## Data Availability

The datasets generated during and/or analyzed during the current study are available in the Surveillance, Epidemiology, and End Results (SEER) repository, Surveillance, Epidemiology, and End Results Program.
