# Supplementary material for: Clinical and Prognostic Factors of Submandibular Gland Malignancies: A SEER-Based Analysis: Analysis of Submandibular Gland Tumors
Source: Head Neck Pathol. 2026 Jan 21;20(1):13. doi: 10.1007/s12105-025-01882-z (PMC12824069; doi:10.1007/s12105-025-01882-z)
Supplement: Supplementary file 1 — Supplementary Material 1 [file 12105_2025_1882_MOESM1_ESM.docx]

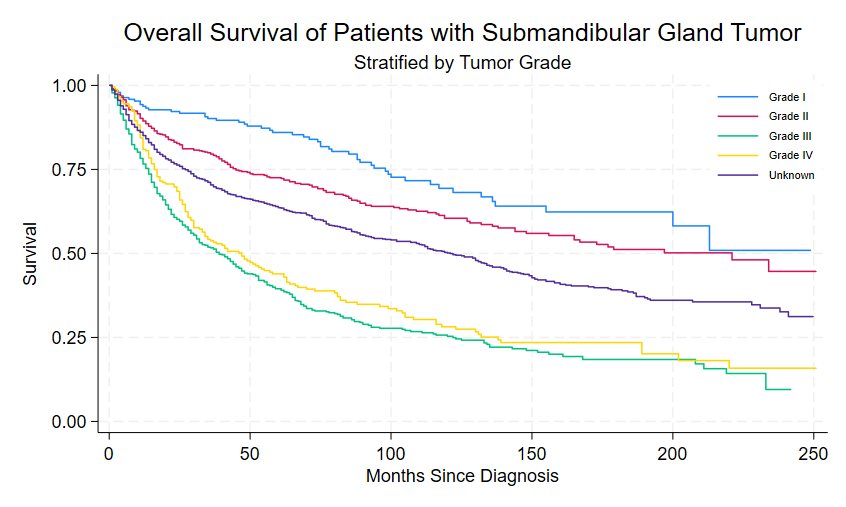


**Figure S1.** Survival outcomes of submandibular gland cancer by tumor grade of differentiation.

**Table S1.** Odds of death by treatment modality, overall and stratified by tumor staging.

*n=2,794 for the overall model. 15 observations were dropped due to missing race/ethnicity. Estimates are adjusted for: age, sex, race/ethnicity, histology subtype, grade, and stage (in the overall model only). The reference category for all estimates is no/unknown treatment. RT=radiation therapy, CTRT=chemotherapy.

|  | Odds of Death (95% CI) | p-value |
| --- | --- | --- |
| Overall | | |
| Surgery Alone | 0.23 (0.17-0.32) | <0.001 |
| Surgery + Adjuvant RT/CTRT | 0.22 (0.16-0.30) | <0.001 |
| Definitive RT/CTRT | 1.15 (0.76-1.72) | 0.511 |
| Other | 0.68 (0.44-1.05) | 0.083 |
| Stages I-II | | |
| Surgery Alone | 0.17 (0.09-0.33) | <0.001 |
| Surgery + Adjuvant RT/CTRT | 0.15 (0.08-0.28) | <0.001 |
| Definitive RT/CTRT | 0.74 (0.27-2.04) | 0.554 |
| Other | 0.38 (0.11-1.33) | 0.129 |
| Stages III-IV | | |
| Surgery Alone | 0.46 (0.25-0.87) | 0.017 |
| Surgery + Adjuvant RT/CTRT | 0.28 (0.16-0.49) | <0.001 |
| Definitive RT/CTRT | 1.43 (0.73-2.79) | 0.297 |
| Other | 0.74 (0.37-1.47) | 0.395 |
